# Therapeutic effect and mechanism of combination therapy with ursolic acid and insulin on diabetic nephropathy in a type I diabetic rat model

**DOI:** 10.3389/fphar.2022.969207

**Published:** 2022-09-30

**Authors:** Yang Liu, Jin-Yan Zheng, Zhi-Tao Wei, Shu-Kun Liu, Ji-Lei Sun, Yin-Hui Mao, Yong-De Xu, Yong Yang

**Affiliations:** ^1^ Department of Urology, The Affiliated Hospital of Changchun University of Chinese Medicine, Changchun, China; ^2^ Department of Endocrinology, The Central Hospital of Zibo, Zibo, China; ^3^ Department of Urology, Beijing Friendship Hospital, Capital Medical University, Beijing, China

**Keywords:** ursolic acid, insulin, diabetic nephropathy, oxidative stress, apoptosis, EMT

## Abstract

This work aims to investigate the therapeutic effect of ursolic acid (UA) plus insulin (In) on diabetic nephropathy (DN) in streptozotocin (STZ)-induced T1DM rats. The experimental groups and operational details are as follows: A total of thirty-two SD rats were divided into four groups: the DN model group (DN, *n* = 8), DN + In treatment group (DN + In, *n* = 8), DN + In + UA administration group (DN + In + UA, *n* = 8), and negative control group (control, *n* = 8). After 8 weeks, changes in renal function indices and pathological damage were assessed. Additionally, oxidative stress-, apoptosis-, and fibrosis-related proteins in kidney tissue were measured. Compared with the control group, the vehicle group showed higher levels of creatine, blood urea nitrogen, urinary protein, apoptosis, and lipid peroxidation; lower superoxide dismutase levels; more severe levels of pathological kidney damage and renal fibrosis; and a deepened degree of EMT and EndMT. Better outcomes were achieved with the combined treatment than with insulin-only treatment. The improvement of TGF-β1, phosphorylated p38 MAPK, FGFR1, SIRT3 and DPP-4 expression levels in renal tissues after combination therapy was greater than that after insulin-only treatment. This study shows that **t**he combination of insulin and UA significantly improved the pathological changes in the renal tissue of T1DM rats, and the underlying mechanism may be related to improving apoptosis and oxidative stress by regulating p38 MAPK, SIRT3, DPP-4 and FGFR1 levels, thereby blocking TGF-β signaling pathway activation and inhibiting EMT and EndMT processes.

## Introduction

Type 1 diabetes mellitus (T1DM), or insulin-dependent diabetes mellitus, is a disorder caused by an absolute deficiency of insulin, causing persistently elevated blood glucose levels and the eventual development of diabetes mellitus (Abraham et al., 2021). The disease occurs mostly in children and adolescents with rapid onset (Morandi et al., 2021). Patients with T1DM are susceptible to DN due to a chronic absolute deficiency of insulin. As one of the most common and devastating microvascular complications of diabetes, DN seriously affects the lives of patients (Gong et al., 2021). When DN shows obvious symptoms, most of them have developed into advanced stage, As the disease continues to progress, the most common symptom is proteinuria. After persistent proteinuria, the glomerular filtration rate decreases, and when the glomerular filtration rate is significantly lower than normal and a large amount of proteinuria appears, it can quickly progress to renal failure stage (Roumeliotis et al., 2021). Its complex pathogenesis is not yet fully understood, and classical views mainly focus on metabolic and hemodynamic changes (Fu et al., 2022). The lack of attention to the disease and its relationship to unhealthy lifestyles has indirectly elevated its incidence. The current clinical treatment plan for T1DM is dominated by lifelong insulin injection, but it requires patient compliance and adherence to healthy lifestyles (Lecumberri et al., 2018).

Renal fibrosis caused by excessive extracellular matrix (ECM) deposition during DN. Fibroblasts generated by activation during epithelial mesenchymal transition (EMT) and endothelial mesenchymal transition (EndMT) are the main source of renal fibroblasts. Renal fibroblasts play an important role in the process of renal fibrosis. There is a complex regulation of the EMT and EndMT processes, and renal fibrosis is the result of the action of multiple signaling pathways and corresponding cytokines, such as the transforming growth factor-β (TGF-β), MAPK, Gh, Wnt, Notch, and Hedgehog signaling pathways (Zhang et al., 2022). Transforming growth factor-β (TGF-β) has also been shown to be a possible fibrogenic factor involved in DN progression (Wu W. et al., 2021; Zheng et al., 2021; Chen et al., 2022). The p38 pathway is one of the MAPK pathways involved in the pathological process of DN (Cui et al., 2019; Gu et al., 2021). Studies have shown that the TGF-β/Smad and MAPK signaling pathways play an important role in the process of renal fibrosis (Geng et al., 2020). In addition, for endothelial fibroblast growth factor receptor 1 (FGFR1) signaling (Xu et al., 2022), the endothelial Sirtuin3 (SIRT3)-mediated signaling mechanism (Srivastava et al., 2021a) and dipeptidyl peptidase-4 (DPP-4)-mediated signaling mechanism (Li et al., 2021) have also become hotspots for DN research.

Currently, there are many therapeutic strategies for DN, but the effectiveness of treatment varies, among which SIRT3, glycolysis inhibitors, DPP-4 inhibitors (e.g., linagliptin), ROCK inhibitors, mineralocorticoid receptor antagonists, and peptide AcSDKP are more widely used in clinical and scientific research to prevent kidney injury (Castoldi et al., 2013; Zhao et al., 2020). For ACEIs and ARBs, blockers of the common renin-angiotensin-aldosterone system (RAAS), the effects in animal models of DN still vary widely (Srivastava et al., 2020a). In this regard, we need to develop a novel therapeutic approach against DN.

Ursolic acid (UA), a pentacyclic triterpene acid compound, is widely found in natural plants and has attracted much attention in recent years (Balcazar et al., 2021). UA has been shown to have antioxidant, anti-inflammatory, antitumor, neuroprotective, hypoglycemic, and other bioactive effects (Zhou et al., 2021; Liu et al., 2022). Some studies have reported that ursolic acid has been used in the treatment of bone injury (Yu et al., 2015), nonalcoholic fatty liver (Li et al., 2014) and cardiovascular diseases (Xiang et al., 2012). At the same time, abundant evidence suggests that hyperglycemia promotes the excessive production of reactive oxygen species (ROS) and oxidative stress (Tota et al., 2021), which accelerates the apoptosis of podocytes (Jin et al., 2019; Qin et al., 2020) and causes renal fibrosis. As a result, it can be hypothesized that UA can exert a therapeutic effect by inhibiting the fibrotic process through anti-EMT and EndMT processes and antioxidant activity.

Therefore, this study investigated the protective effect of UA combined with insulin on renal injury in type 1 diabetic rats and its mechanism by establishing an STZ-induced type I diabetic rat model to provide a theoretical basis for the clinical application of UA on DN.

## Materials and methods

### Laboratory animals

Thirty-two 8-week-old male SPF-grade SD rats, weighing 210–240 g, were purchased from the Laboratory Animal Center of Changchun University of Chinese Medicine [Medical Experimental Animal Number SYXK (Ji)2018-0014], and all animal experiments were approved by the Ethics Committee of Changchun University of Chinese Medicine on 6 January 2020. (Changchun, China; Approval No. 2020132). The animals were given a standard pellet diet and tap water, and the experiments began after 1 week of adaptive feeding without abnormalities.

### Establishment of the DN rat model

Thirty-two SD male rats were randomly divided into a negative control group (*n* = 8) and a modeling group (*n* = 24). STZ (HPLC ≥98%, Sigma, United States) solution was prepared as follows (Zheng et al., 2020): 1 g of STZ was dissolved in 100 ml of citric acid-sodium citrate buffer (0.1 mol/L, pH = 4.5) in an ice bath to a final concentration of 10 mg/ml; this ready-to-use solution was kept dry and protected from light. Before formal modeling, all rats were weighed, random blood glucose was measured, and the T1DM model was induced after 12 h of fasting without water. The T1DM model was induced by a single intraperitoneal injection of streptozotocin (STZ, 60 mg/kg) in SD rats, and the negative control group was injected intraperitoneally with an equal amount of citrate-sodium citrate buffer. Random blood glucose and body weight measurements were performed daily after injection, and rats with blood glucose >16.67 mmol/L for 3 days, accompanied by obvious symptoms of polyuria, excessive drinking, and excessive eating, were considered successful.

### Experimental grouping and treatment

After 4 weeks of modeling, the rats in the model group were rerandomized. The final DM rats were randomly divided into three groups: DN model group (DN, n = 8), DN + insulin treatment group (DN + In, n = 8), DN + In + UA administration group (DN + In + UA, *n* = 8), and another negative control group (control, *n* = 8). Rats in the insulin group were given an intraperitoneal injection of glargine insulin (Aventis Pharma, Germany, 1–3 U/100 g) once in the morning and once in the evening, while rats in the UA and insulin combination group were given an intraperitoneal injection of insulin along with UA (HPLC ≥98%, Absin, Shanghai, China, 20 mg/ml) (solvent: DMSO:ddH2O = 1:9, 50 mg/kg) gavage, and all groups were given an equal amount of solvent gavage once daily for 4 weeks. At the end of the study, diabetic rats received approximately 10–12 U/day of insulin based on daily glucose changes.

### General index testing

The body weight and tail vein glucose level of each rat were measured before and after modeling and after the last dose of treatment, as were the 24-h diet and water intakes of each group of rats. At the end of the experiment, the rats were anesthetized by intraperitoneal injection of 3% sodium pentobarbital (30 mg/kg), and the right kidney was rapidly removed after the rats were completely anesthetized. Finally, they were euthanized by cervical dragging. The kidney weight was measured, and the kidney index was calculated according to the last weight (kidney mass/body mass).

### Testing of biochemical indicators of renal function

During the experiment, metabolic cages were used to collect 24-h urine from rats. Before the rats were euthanized, 4 ml of blood was collected from the carotid artery and centrifuged at 3,500 r/min and 4°C for 20 min. The serum was collected and centrifuged at 4°C (3,500 r/min, 10 min); the supernatant was stored at −20°C for measurement. Serum creatinine (SCr), blood urea nitrogen (BUN), and urine protein content were measured by an automatic biochemical analyzer (Lei Du lives Science and Technology Limited Company, Shenzhen, China).

### Renal pathology staining

The kidney tissues of rats in each group were fixed with 4% paraformaldehyde, embedded in paraffin, and routinely sectioned at a thickness of 5 μm. These sections were dehydrated in ethanol, cleared in xylene, dewaxed in xylene, stained with HE, periodic acid-Schiff (PAS), Sirius red and Masson trichrome stained (MTS), dehydrated, cleared, sealed, and photographed microscopically, and morphological changes in the kidney tissues were recorded. A point-counting method on a microscopic grid was used to assess the relative area of each rat glomerular thylakoid stroma in PAS staining. The area of fibrosis in Sirius red- and MTS-stained tissue images was assessed using ImageJ software. For each rat kidney, images of six different fields of view (×40) were randomly evaluated.

### Oxidative stress indicator test

An appropriate amount of kidney tissue was removed, and the 10% homogenate was placed in an ice bath to be centrifuged at 4°C (4,000 rpm/min) for 10 min; the supernatant was stored at −20°C. The expression levels of superoxide dismutase (SOD) and malondialdehyde (MDA) in kidney tissues were measured according to the kit instructions (Beyotime Biotechnology, Shanghai, China).

### TUNEL fluorescent staining

A reaction solution was added for staining according to the TUNEL kit instructions (Roche, Switzerland). The nuclei were stained blue; positive cells were stained green under the microscope to calculate the apoptotic rate. Apoptotic cells (green) were counted at 200×; six unconnected fields of view were selected for each group to calculate the apoptotic rate as follows: apoptotic rate (%) = (number of TUNEL-positive cells/total number of cells) × 100%.

### Immunofluorescence staining

Frozen kidney sections (5 μm) were used for immunofluorescence to express EndMT and EMT levels. Positive markers for α-smooth muscle actin (α-SMA, Boster, BM0002, 1:500), Vimentin (Servicebio, GB11192, 1:300), E-cadherin (Proteintech, 20874-1-AP, 1:200) and CD31 (Abcam, ab281583, 1:200) were assessed. Briefly, the frozen sections were first dried, washed 3 times (5 min) in PBS solution, and blocked with 2% bovine serum albumin (BSA)/PBS for 30 min at room temperature. Thereafter, specimens in sections were incubated in primary antibody for 90 min, followed by three washes of sections in PBS for 5 min each. Next, the specimens were incubated with secondary antibody for 40 min and washed again 3 times in PBS (5 min each time). Finally, the sections were sealed with a DAPI-containing blocking liquid. Immunolabeled sections were analyzed by fluorescence microscopy. For each kidney section, pictures of six different areas (×400) were taken and analyzed quantitatively.

### Immunohistochemical testing

Paraffin sections were dewaxed, hydrated and repaired with high-pressure antigen heat. The sections were sealed and incubated with antibody (Caspase-3:Proteintech,19677-1-AP,1:200; Bax: BOSTER,BA0315-1,1:200; FGFR1:Cell Signaling Technology,#9740,1:500; SIRT3:affinity: AF5135, 1:100; DPP-4: Abcam, ab187048, 1:2000; TGF-β1, Abcam, ab50036,1:200) at 4°C overnight, washed, incubated with secondary antibody at 37°C for 1 h, and washed for DAB color development. Six fields of view were randomly selected from each section under the microscope and saved by video; the macro program for positive expression was set up using ImageJ software to analyze the expression of target proteins in different groups under the same conditions.

### Western blotting for protein expression in kidney tissues

Kidney tissues were lysed in RIPA buffer (Beyotime Biotechnology, Shanghai, China); protein concentrations were determined using the BCA Protein Assay Kit (Beyotime Biotechnology). Equal amounts of proteins were resolved by SDS‒PAGE, and the proteins were transferred to PVDF membranes and incubated with the appropriate antibodies.

Rabbit anti-rat TGF-β1 antibody (Abcam, ab50036, 1:1,000), phospho-p38 MAPK antibody (Thr180/Tyr182) (Cell Signaling Technology, #4511, 1:1,000), p38 MAPK antibody (Cell Signaling Technology, #8690, 1:1,000) and GAPDH internal reference antibody (Millipore, #3241215, 1:10,000) were added and incubated overnight at 4°C. Development was performed according to the ECL kit instructions, and the protein bands were quantified in grayscale values using Gel EQ Quantity One software (Bio-Rad, United States).

### Statistical analyses

SPSS 26.0 statistical software (SPSS Inc., Chicago, IL, United States) was used to analyze the statistical significance of the data. ImageJ software (National Institutes of Health, Bethesda, MD, United States) was used for quantitative analysis of the data. One-way ANOVA was used for comparisons between multiple groups, and Tukey’s HSD method was used for two-way comparisons. *p* < 0.05 indicated that the differences were statistically significant.

## Results

### Comparison of blood glucose in rats

**FIGURE 1:**
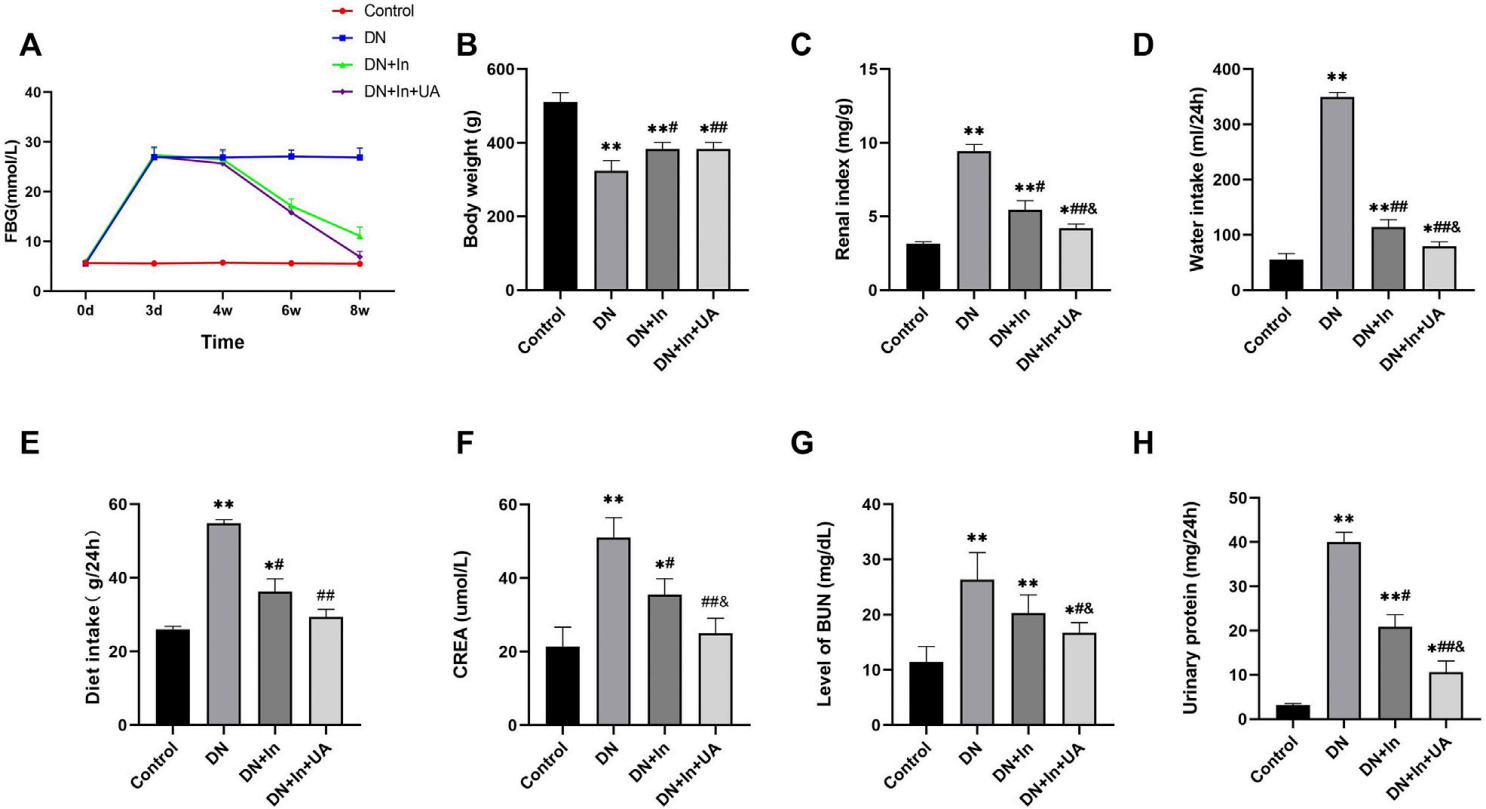
General condition of the rats. **(A)** Effect of ursolic acid and insulin on fasting blood glucose levels in diabetic rats. Fasting blood glucose (FBG) levels were correlated with once daily oral UA (50 mg/kg) for a 4-week duration of treatment. **(B–E)** Comparison of the final body weight, renal index, and total daily diet and water intake of the four groups of rats in the final week. **(F–H)** Comparison of blood creatinine, blood urea nitrogen and urine protein levels in the four groups of rats. Data are expressed as the mean ± S.D. (*n* = 6). *p < 0.05, **p < 0.01 vs. the control group; #*p* < 0.05, ##*p* < 0.01 vs. the DN group; and *p* < 0.05 vs. the DN + In group. Control, control group; DN, diabetic nephropathy model group; DN + In, insulin treatment group; DN + In + UA, insulin and ursolic acid combination therapy group.

The fasting blood glucose levels of the rats were monitored at 0 h, 72 h, 4, 6 and 8 weeks after 12 h of fasting. FBG was significantly increased in the DN group and maintained at a higher level throughout the experimental period. FBG was significantly lower in the insulin-treated group alone than in the DN group (*p* < 0.01) and was even more significant after the combination of ursolic acid and insulin (*p* < 0.01). These results suggest that UA has a hypoglycemic effect on DN (Figure 1A).

### General condition of the rats

By the end of the experiment, there were two dead rats in the DM group and one dead rat in the DM + In group. Compared with the negative control rats, the body weight and renal index of DN rats were significantly higher (*p* < 0.01), indicating that the kidneys of DN rats showed hypertrophy. In contrast, the renal index was significantly lower in both the UA and insulin-treated groups than in the DN group (*p* < 0.01), suggesting that UA could have an ameliorative effect by reducing renal hypertrophy in DN rats (Figures 1B,C). Compared with the negative control rats, the DN rats came out with significantly more food and more drink (*p* < 0.01). In contrast, DN + In + UA treatment groups were more convergent to the normal group rats compared with the DN group (*p* < 0.01), suggesting that UA could alleviate the clinical manifestations of diabetes in the DN rats (Figures 1D,E).

### Comparison of renal function and biochemical parameters in rats

Compared with the control group, rats in the DN model group showed significant differences in SCr, BUN and urinary protein amount (all *p* < 0.01); compared with levels in the DN model group, the SCr (*p* < 0.01), BUN (*p* < 0.05), and urinary protein volume (*p* < 0.01) were significantly improved in the combined insulin and UA group compared with the DN model group. There was a significant difference between the SCr, BUN and urine protein levels in the DN + In group and the DN + In + UA combination group (*p* < 0.05) (Figures 1F–H).

### Morphological evaluation of the rat kidney

**FIGURE 2:**
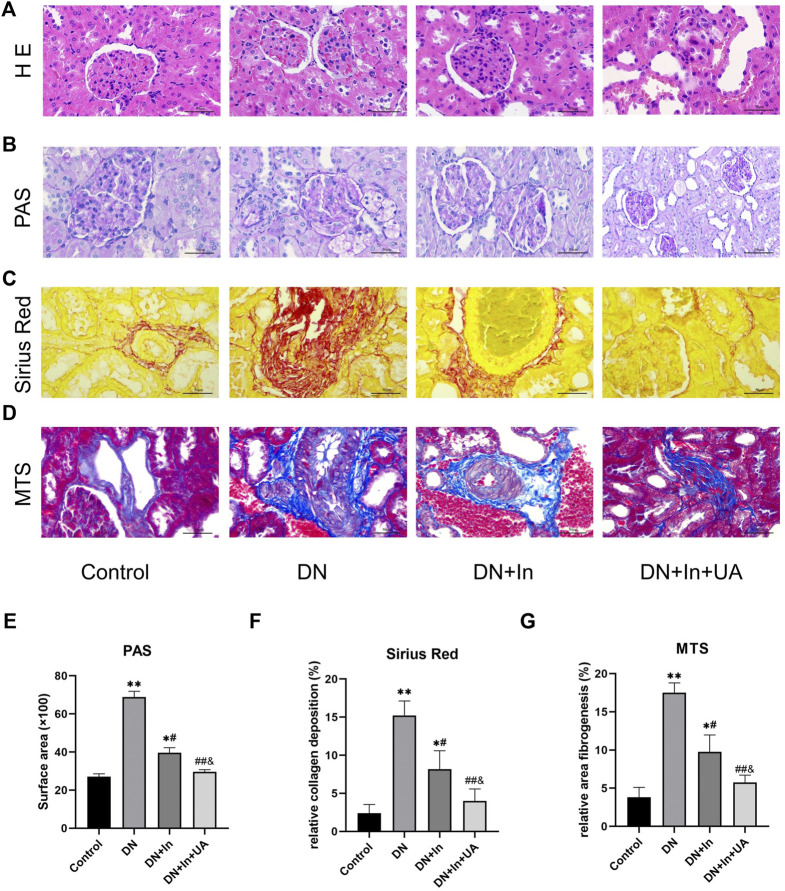
Morphological observation of rat kidney. **(A)** Representative images of HE staining of pathological structures of rat kidney. **(B)** PAS, **(C)** Sirius red, **(D)** MTS in the control, DN-, DN + In- and DN + In + UA-treated diabetic kidneys. **(E–G)** Quantitative analysis of PAS, Sirius red, and MTS staining in the four groups of rats. Magnification: ×400, scale bars = 50 μm. Data are expressed as the mean ± S.D. (*n* = 5). *p < 0.05, **p < 0.01 vs. the control group; #*p* < 0.05, ##*p* < 0.01 vs. the DN group; and *p* < 0.05 vs. the DN + In group.

Based on HE staining, PAS staining, Sirius red staining and MTS, no pathological changes were observed in the kidney tissue of the control group, while the glomerular basement membrane was thickened, the thylakoid matrix was hyperplastic, and glomerulosclerosis was obvious in the DN model group. Compared with the DN model group, the glomerular basement membrane remained thickened, the thylakoid matrix was mildly hyperplastic in the insulin group, and the degree of glomerulosclerosis was reduced; in the UA and insulin combination group, however, the degree of lesion was significantly reduced and normalized (Figures 2A–D). Meanwhile, quantitative analysis of their staining results revealed that fibrosis levels were significantly higher in the DN group than in the control group (*p* < 0.01), and after treatment, fibrosis improved more significantly in the DN + In + UA group (*p* < 0.01) and was statistically significant with the DN + In group (*p* < 0.05) (Figures 2E–G).

### Oxidative stress injury in the rat kidney

**FIGURE 3:**
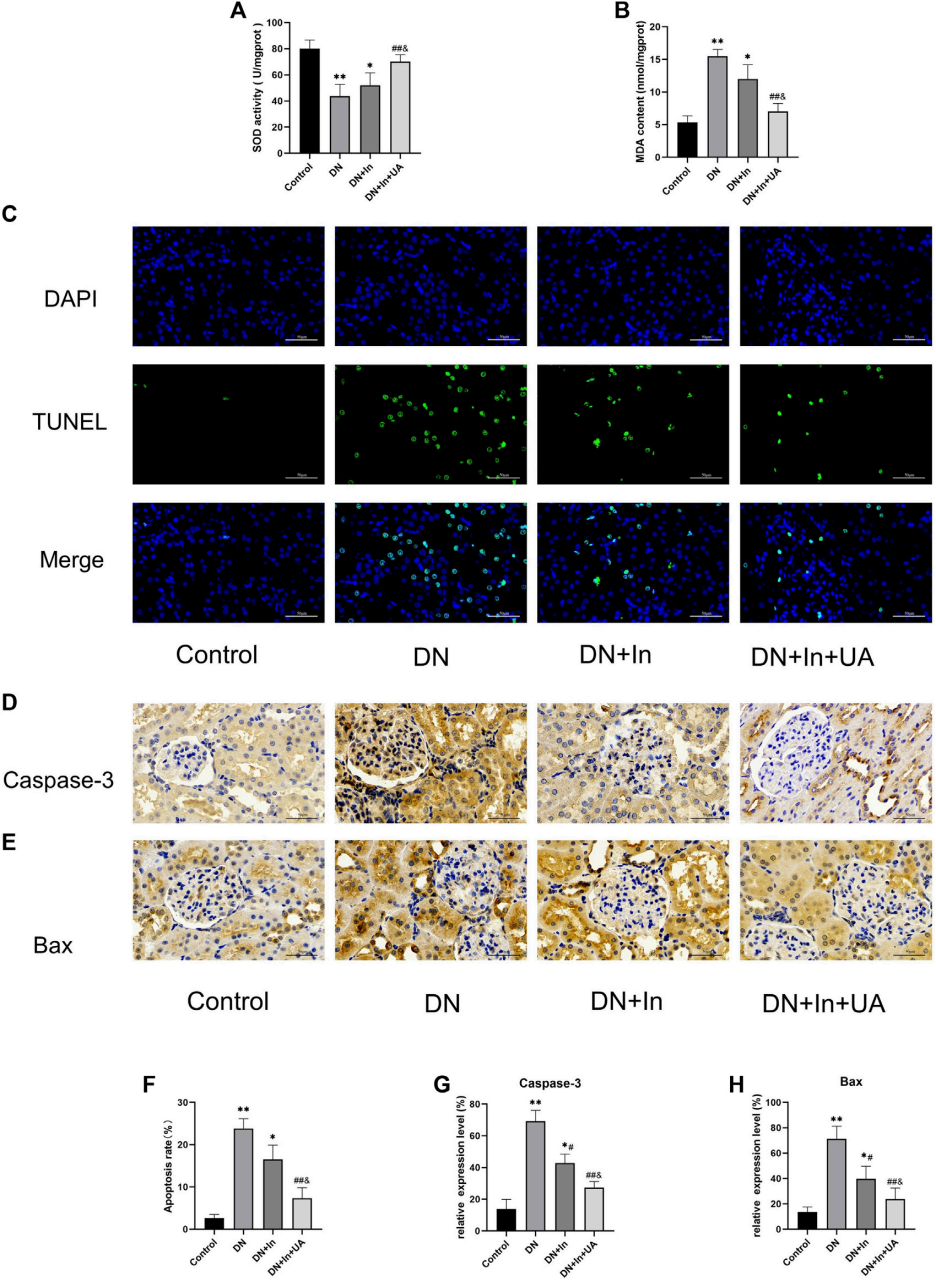
Detection of oxidative stress levels and apoptosis indicators in kidney tissues. **(A,B)** The kidney oxidative stress index of rats in each group. **(C)** TUNEL fluorescence staining to detect apoptosis levels in kidney tissue. The nucleus is in blue, and the apoptotic cells are in green. The bottom picture is a composite of both. **(D,E)** Characteristic images of immunohistochemical staining for Caspase-3 and Bax in kidney tissue (×400, scale bars = 50 μm). **(F)** Semiquantitative statistical graph of the percentage of apoptotic cells. **(G,H)** Quantitative analysis of Caspase-3 and Bax expression levels in kidney tissues from the four groups. Magnification: ×400, scale bars = 50 μm. Data are expressed as the mean ± S.D. (n = 6). *p < 0.05, **p < 0.01 vs. the control group; #*p* < 0.05, ##*p* < 0.01 vs. the DN group; and *p* < 0.05 vs. the DN + In group.

In the DM model group, the MDA content in the kidney tissue of rats was significantly increased compared with that in the control group, and SOD activity was significantly decreased (both *p* < 0.01). After treatment, the MDA content in the kidney tissue of rats in the UA + insulin group was significantly decreased, and SOD activity was significantly increased (both p<0.01 ). Relative to the MDA content and SOD activity expression in the DN + In group, there was a significant difference in the DN + In + UA group (*p* < 0.05) (Figures 3A,B).

### Detection of apoptosis levels in rat kidney tissues

Renal apoptosis in rats was observed by TUNEL staining (Figures 3C,D). The degree of apoptosis was significantly increased in the DN group of rats compared to the control group (*p* < 0.01). Interestingly, after treatment in the DN + In + UA group, the level of TUNEL-positive cells was significantly reduced (*p* < 0.01), and the level of TUNEL-positive cells in the DN + In + UA group was less than that in the DN + In group (*p* < 0.05).

The expression of the apoptosis-related proteins caspase-3 and Bax was observed by immunohistochemistry (Figure 3E). The expression of caspase-3 (Figure 3F) and Bax (Figure 3G) proteins was greatly increased (*p* < 0.01) in the DN group rats compared to the control group, indicating more severe renal apoptosis. Reassuringly, treatment in the DN + In + UA group significantly reversed the expression of caspase-3 and Bax proteins compared with rats in the DN group (*p* < 0.01). Notably, the expression levels of caspase-3 and Bax proteins were reduced in the DN + In + UA group compared to the DN + In group (*p* < 0.05).

### EMT and EndMT expression

**FIGURE 4:**
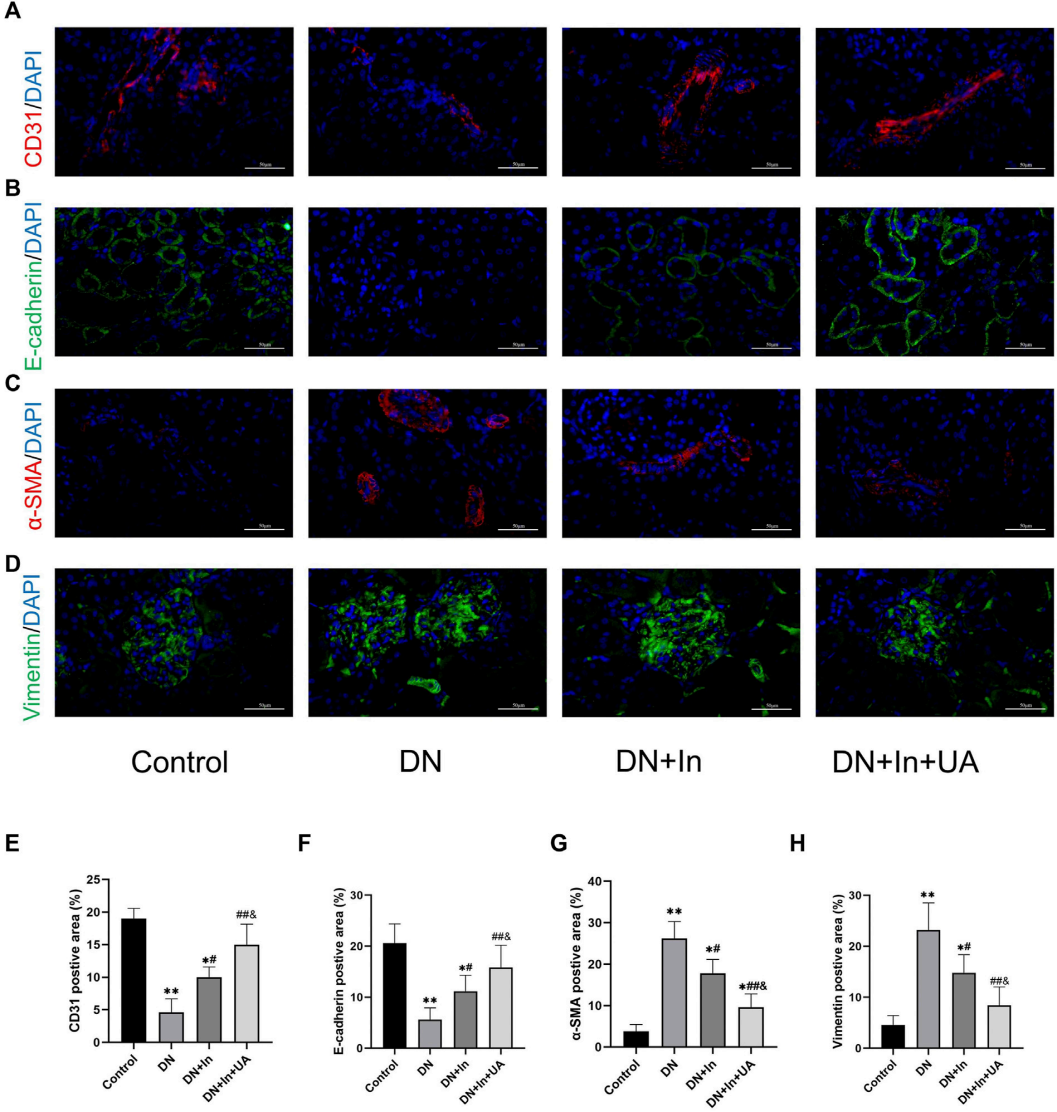
EMT and EndMT analysis in kidney tissues. Typical images of the endothelial cell marker **(A)** CD31, the epithelial cell marker **(B)** E-cadherin, and the mesenchymal cell markers **(C)** α-SMA and **(D)** Vimentin. **(E–H)** Quantification of CD31, E-cadherin, α-SMA and Vimentin in diabetic kidney tissues treated with control, DN, DN + In and DN + In + UA. Decreases in the epithelial marker E-cadherin and the endothelial marker CD31, along with increases in the mesenchymal marker α-SMA and Vimentin, were considered to be undergoing EMT and EndMT. Magnification: ×400, scale bars = 50 μm. Data are expressed as the mean ± S.D. (*n* = 5). *p < 0.05, **p < 0.01 vs. the control group; #*p* < 0.05, ##*p* < 0.01 vs. the DN group; and *p* < 0.05 vs. the DN + In group.

In the DN group of rats, we found that the endothelial cell marker CD31 (Figure 4A) and the epithelial cell marker E-cadherin (Figure 4B) were significantly reduced (*p* < 0.01). CD31 and E-cadherin expression levels were significantly higher in both the DN + In group and DN + In + UA group than in the DN group (*p* < 0.05; *p* < 0.01). Notably, CD31 and E-cadherin expression levels in the DN + In + UA group remained statistically significant for the DN + In group (*p* < 0.05). Meanwhile, the MSC markers α-SMA (Figure 4C) and Vimentin (Figure 4D) were significantly elevated in the DN group (*p* < 0.01) and significantly decreased after ursolic acid and insulin treatment (*p* < 0.01) but remained statistically significant compared to the DN + In group (*p* < 0.05). It is generally accepted that the epithelial cell marker E-cadherin is associated with a decrease in the endothelial cell marker CD31 and an increase in the mesenchymal cell marker α-SMA and Vimentin is considered to be undergoing EMT and EndMT (Figures 4E–H).

### Expression of TGF-β1 in kidney tissues

**FIGURE 5:**
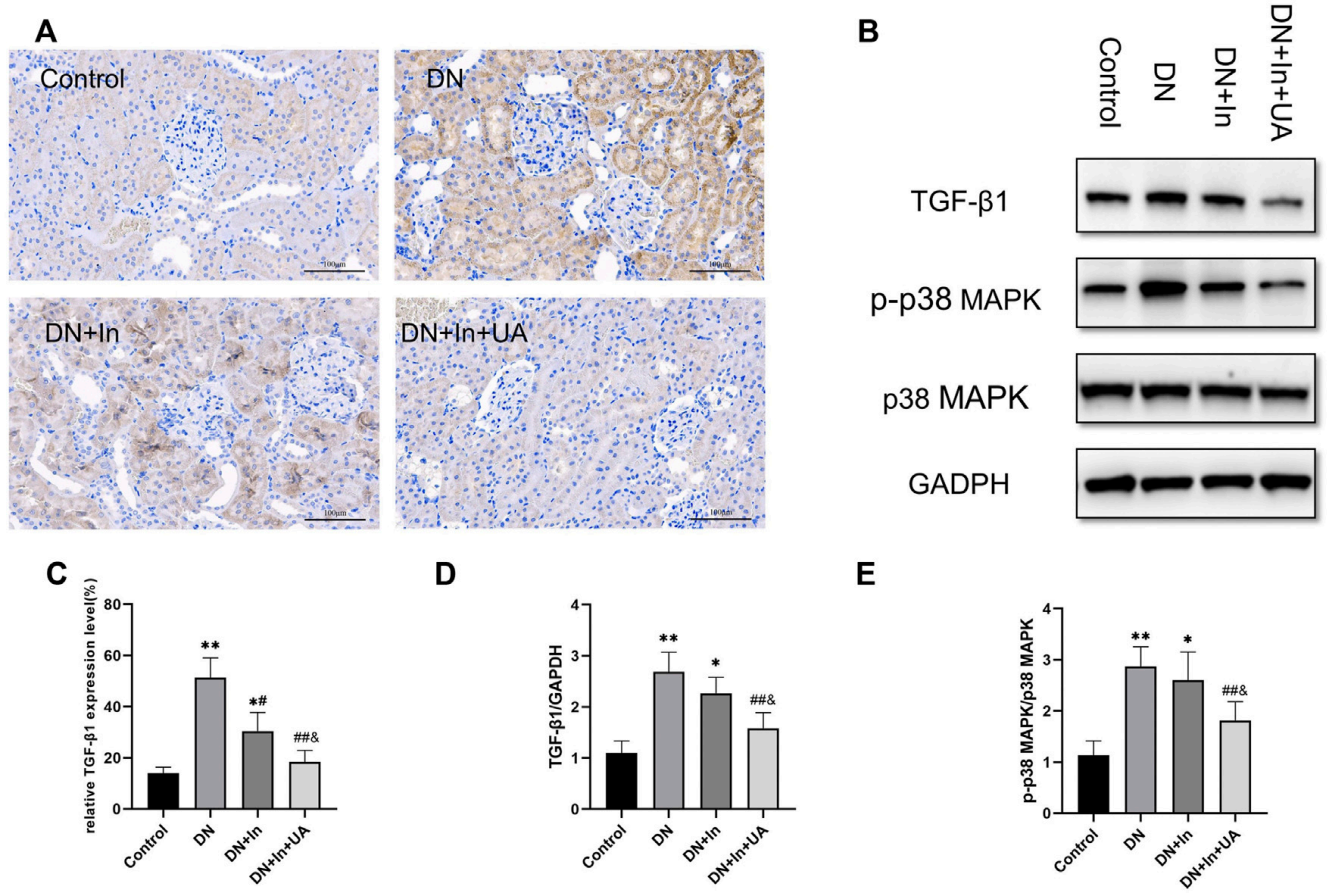
Expression of TGF-β1 and p38 signaling factors. **(A)** TGF-β1 expression as assessed by immunohistochemical staining of the kidney. Positive expression of TGF-β1 appears as dark brown granules in the cell membrane and/or cytoplasm (magnification: ×200, scale bars = 100 μm). **(B)** Western blot detection of TGF-β1, p-p38 MAPK and p38 MAPK expression in each group. **(C)** Semiquantitative analysis of the positive expression of TGF-β1 in immunohistochemistry. **(D,E)** Quantitative analysis of TGF-β1 and p-p38 MAPK expression in kidney tissues in Western blot experiments. Data are expressed as the mean ± S.D. (*n* = 6). *p < 0.05, **p < 0.01 vs. the control group; #*p* < 0.05, ##*p* < 0.01 vs. the DN group; and *p* < 0.05 vs. the DN + In group.

In immunohistochemical staining, the TGF-β1 expression level was significantly higher in the kidney tissue of the DN model group than in the control group; UA combined with insulin treatment significantly reduced the TGF-β1 expression level (*p* < 0.01). There was a significant difference between the DN + In + UA group and the DN + In treatment group (*p* < 0.05). In the western blot assay, the expression level of TGF-β1 in the kidney tissue of rats in the DN model group was significantly higher than that in the control group (*p* < 0.01); DN + In + UA treatment significantly reduced the expression level of TGF-β1 (*p* < 0.01), and the levels in the DN + In + UA treatment group and the DN + In treatment group were significantly different from each other ( p<0.05 ) (Figures 5A,C).

### Western blot detection of p38 MAPK expression in kidney tissues

The level of p-p38 MAPK expression in the kidney tissue of rats in the model group was significantly higher than that in the control group (*p* < 0.01). UA combined with insulin treatment reduced the level of p38 MAPK phosphorylation (*p* < 0.01). There was a statistically significant difference between the combined treatment and the insulin alone groups, which were significantly different from each other (*p* < 0.05). P38 MAPK expression was not significantly different between the groups (*p* > 0.05) (Figures 5B,D,E).

### Exploration of classical signaling pathways

**FIGURE 6:**
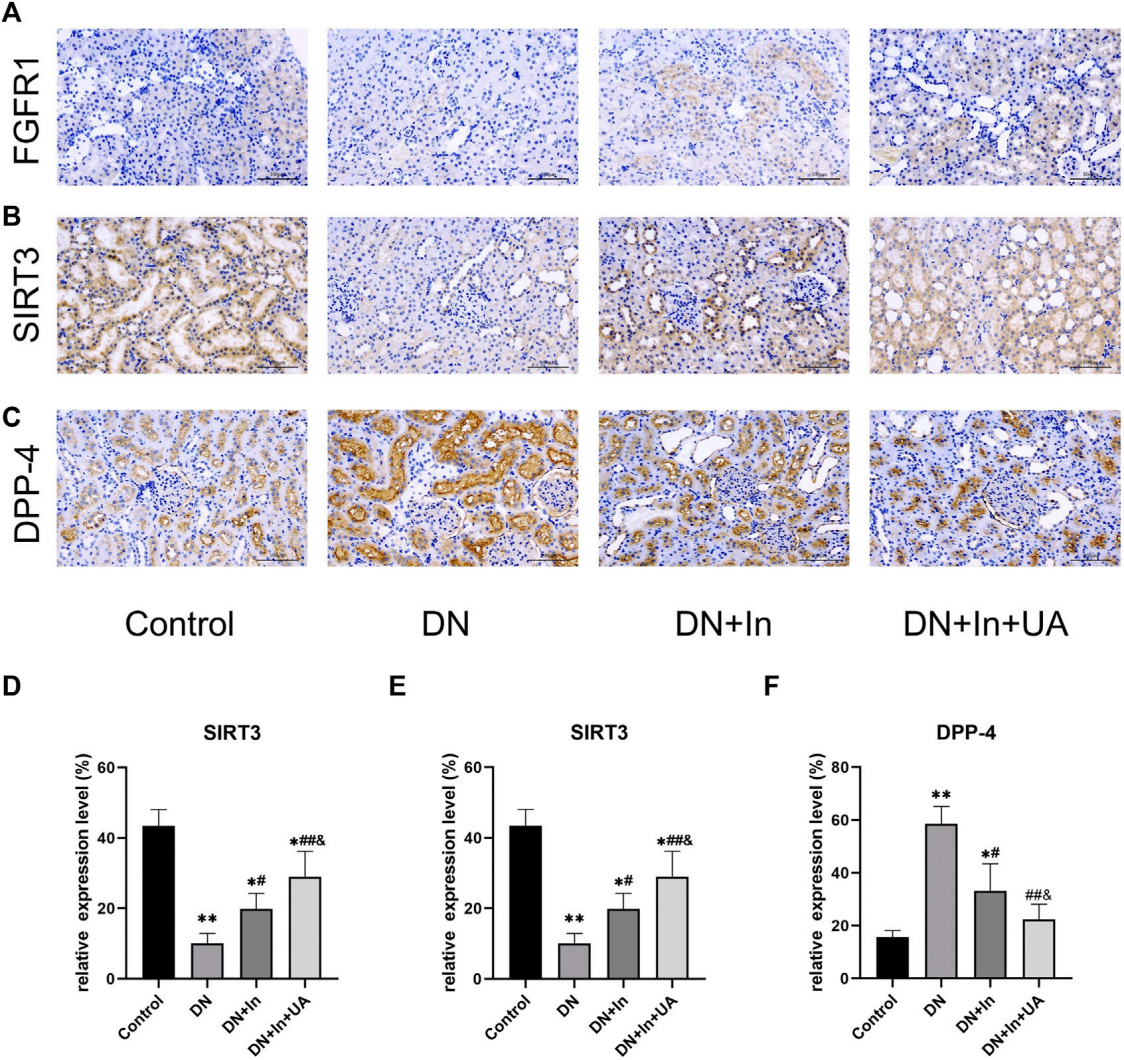
Exploration of classical signaling pathways. The expression levels of **(A)** FGFR1, **(B)** SIRT3, and **(C)** DPP-4 in kidney tissues were examined by immunohistochemical staining. **(D–F)** Quantitative analysis of FGFR1, SIRT3 and DPP-4 expression levels in renal tissues of each group. Magnification: ×200, scale bars = 100 μm. Data are expressed as the mean ± S.D. (*n* = 5). *p < 0.05, **p < 0.01 vs. the control group; #*p* < 0.05, ##*p* < 0.01 vs. the DN group; and *p* < 0.05 vs. the DN + In group.

The protein expression levels of FGFR1 (Figure 6A), SIRT3 (Figure 6B), and DPP-4 (Figure 6C) were examined by immunohistochemical staining in kidney tissues. We found that the expression levels of FGFR1 and SIRT3 were significantly lower in the DN group than in the control group (*p* < 0.01). After combined treatment with ursolic acid and insulin, FGFR1 and SIRT3 expression levels showed a significant increase (*p* < 0.01) and remained higher than those in the DN + In group (*p* < 0.05) (Figures 6D,E). In contrast, DPP-4 expression levels in kidney tissues showed the opposite trend. (Figure 6F).

## Discussion

Current treatment strategies for DN include glycemic and blood pressure control, a low-protein diet, lipid-lowering drugs, and interference with the renin-angiotensin (RAS) system (Ahola et al., 2021; Zhang and Jiang, 2022). Despite their efficacies, most patients still develop end-stage renal disease, and the lethality rate remains high. Therefore, better treatments and interventions are needed.

Our experiment showed classic clinical symptoms of “excessive drinking, polyphagia, polyuria, and weight loss” in DN rats, which significantly improved after treatment. We also detected a significant increase in the levels of SCr and BUN, indicating severely impaired renal function, which further elevated 24-h urinary protein in the urine. This is generally considered to be the initial step in DN, from normoalbuminuria to microalbuminuria (Ramaphane et al., 2021). It is well documented that good glycemic control improves microalbuminuria and reduces the risk of nephropathy in diabetic patients (de Boer et al., 2011). After 4 weeks of therapeutic intervention, all treatment groups showed significantly lower indices than those of the model group, and the treatment effect was more effective in the insulin plus UA combination group. This result was also consistent with the results reported by Ma et al. (Mapanga et al., 2009), suggesting that UA has a protective effect on DN renal tissue. DN is characterized by morphological and ultrastructural changes in the kidney. HE, Sirius red, PAS and Masson staining revealed pathological manifestations in DN rats, such as glomerular hypertrophy, a thickened glomerular tubular basement membrane dense layer, glomerular nodular sclerosis and tubulointerstitial fibrosis. After combined treatment with insulin and UA, the above pathological changes were partially reversed. Compared with the insulin group, the combination treatment group had a significant therapeutic effect, which also visually demonstrated the protective effect of UA on DN renal tissue.

We know that the relevant features of STZ-induced animals are similar to the clinical features of human diabetes (Westenfelder et al., 2021). High blood glucose can cause the production of ROS and lipid peroxidation, which can trigger DN (Dai et al., 2021). The aggravation of DN further leads to the aggregation of ROS, which generate lipid peroxides (e.g., MDA), leading to the oxidation of proteins and amino acids to aggravate the progression of the disease. In a basic study, Tong et al. (Tong et al., 2019) demonstrated that ethyl vanillin could protect against the progression to DN from kidney injury by inhibiting oxidative stress and apoptosis, which provided us with ideas for studying the mechanism of UA treatment. The results of the basic experiments conducted by Wang *et al.* showed that UA ameliorated oxidative stress, inflammation and fibrosis in rats with diabetic cardiomyopathy (Wang et al., 2018). The results of this study showed that the SOD levels of DN rats were all significantly reduced, and the lipid peroxide index (the MDA content) was significantly increased. The changes in the MDA and SOD contents reflected the enhanced level of oxidative stress in the kidney tissue of the model group rats, and the above phenomenon was reversed after treatment with insulin and UA, which indicated that UA not only lowered blood glucose but also had good antioxidant ability. Data from the study by Xu et al. further suggest that UA can be used as a protective agent against renal dysfunction through its antioxidant and anti-inflammatory effects (Xu et al., 2018).

In a rat model of diabetes induced by a high-sugar, high-fat diet and STZ intraperitoneal injection, investigators using a combination of UA and other drugs for DN found that its protective effects on the kidney may be related to oxidative stress, renal fibrosis, and anti-apoptosis (Wu et al., 2021b). In this experiment, it was concluded by TUNEL fluorescence staining and expression of Caspase-3 and Bax in immunohistochemistry that apoptosis was increased in the kidney of rats with DN and was significantly reduced after the application of insulin combined with UA treatment, so it is presumed that UA has good anti-apoptotic ability to repair the kidney functional damage in T1DM rats.

In DN, ECM deposition results in renal fibrosis. Fibroblasts produced by activation during EMT and EndMT are the main source of renal fibroblasts. In the present experiment, in the DN group, the decrease in the epithelial cell marker E-cadherin with the endothelial cell marker CD31 and the increase in the mesenchymal cell markers α-SMA and Vimentin indicated that the process of EMT and EndMT was being undergone, and this was improved after treatment. The study shows that TGF-β1 can stimulate the excessive accumulation of ECM through EMT, which leads to renal fibrosis (Gwon et al., 2021). TGF-β1 is involved in renal cell hypertrophy, proliferation and apoptosis in addition to ECM protein synthesis (Zhang et al., 2021). In the present study, the expression level of TGF-β1 was significantly elevated in DN rats, which is consistent with the findings of Barbara et al. in db/db mice (Toffoli et al., 2020). After combined treatment with insulin and UA, the expression level of TGF-β1 was reduced, suggesting that UA may improve the extent of renal fibrosis by reducing TGF-β1 expression. A clinical study by Mumtaz *et al.* also found elevated levels of TGF-β1 expression in DN patients (Takir et al., 2016), which has important implications for the subsequent treatment of DN. The p38 MAPK signaling pathway increases the release of inflammatory mediators by increasing ROS production, regulates the RAS system, affects glomerular thylakoid extracellular matrix formation and degradation, and can be activated by high glucose to accelerate DN (Susztak et al., 2006). In early DN, there are increased levels of p38 MAPK phosphorylation in the glomerulus (Kang et al., 2001). In the western blot assay, the same results were obtained wherein the p-p38 MAPK expression level was significantly increased in the DN group rats; after treatment, the p-p38 MAPK level was able to converge to that of the negative control rats, showing that UA seems to affect the transduction of this signaling pathway during the treatment of DN rats. A number of novel drugs for DN treatment have been shown to affect the expression of p-p38 MAPK and TGF-β1 to improve renal tubulointerstitial fibrosis in experimental animals with DN (Fujita et al., 2004; Cheng et al., 2013; Jiang et al., 2017; Wang et al., 2019a).

There are many ways to treat DN, among which glucagon-like polypeptide-1 (GLP-1) receptor agonists can slow down the progression of DN. DPP-4 inhibitors reduce GLP-1 inactivation by inhibiting the activity of DPP-4 and are currently used in kidney injury due to type 2 diabetes. In our experiment, rats in the DN group also showed an increase in the activity of DPP-4, and the level of DPP-4 expression was significantly reduced after combined treatment with ursolic acid and insulin. A study showed that treatment of rats with DN with linagliptin, a DPP-4 inhibitor, reduced proteinuria and slowed fibrotic kidney damage without affecting blood glucose levels (Sharkovska et al., 2014). Sodium glucose cotransporter 2 (SGLT2) inhibitors are beneficial in the prevention of DN. SGLT2 inhibitors exhibit renoprotective potential that relies in part on inhibition of glucose reabsorption and subsequent aberrant glycolysis in the renal tubules (Li et al., 2020b). Mineralocorticoid receptor antagonist (MRA) can block sodium reabsorption and overactivation of mineralocorticoid receptor (MR) in renal epithelial or vascular tissues mediated by MR, avoiding MR overactivation leading to fibrosis and inflammation and thus protecting the function of the kidney, which has a good prognosis for patients with chronic kidney disease (Oka et al., 2022). It has been shown that diabetes accelerates renal fibrosis in mice lacking the endothelial glucocorticoid receptor (GR) compared to control mice (Srivastava et al., 2021c). These data demonstrate that loss of podocyte GR leads to upregulation of Wnt signaling and disruption in fatty acid metabolism. Podocyte–endothelial cell crosstalk, mediated through GR, is important for glomerular homeostasis, and its disruption likely contributes to DN. It is well documented that deletion of podocyte GR leads to upregulation of Wnt signaling and disruption of fatty acid metabolism, which are important factors that may contribute to DN (Srivastava et al., 2021b).

Although novel hypoglycemic agents, such as GLP-1 analogs, SGLT2 inhibitors, and DPP-4 inhibitors, have made great progress in the protective effects of DN, they still need to be used in combination with RAAS blockers. ACEIs and ARBs are not only effective in controlling blood pressure and reducing urinary protein in the clinical management of early-to mid-stage DN but also in delaying the risk of progression of chronic kidney disease to end-stage renal disease (Deng et al., 2022). In a diabetic mouse model, ACEIs were observed to ameliorate renal fibrosis by attenuating DPP-4 and TGF-β signaling but were not demonstrated by ARBs. Furthermore, the combination of N-acetyl-serinyl-aspartyl-lysinyl-proline (AcSDKP), one of the ACE substrates, with ACEI slightly enhanced the inhibitory effect of ACEI on DPP-4 and related TGF-β signaling and revealed miR-29s and miR-let-7s as key antifibrotic players. Interestingly, ACEIs also restored miR-29 and miR-let-7 family crosstalk in endothelial cells, suggesting that the antifibrotic effects of ACEIs are due to AcSDKP-mediated antifibrotic mechanisms (Srivastava et al., 2020a). This mechanism reprograms central metabolism, including restoration of SIRT3 protein and mitochondrial fatty acid oxidation and inhibition of abnormal glucose metabolism in DN. Inhibition of AcSDKP leads to disruption of renal cellular metabolism and activation of interstitial transformation, leading to severe fibrosis in the diabetic kidney (Srivastava et al., 2020b). In our experiment, it was also found that the SIRT3 protein expression level was significantly reduced in the DN group rats, which was significantly improved by the combined treatment of ursolic acid and insulin. Wang et al. (Wang et al., 2019) found that activation of SIRT3 induced mitochondrial biosynthesis and prevented renal oxidative stress and lipid accumulation through *in vitro* and *in vivo* studies. Endothelial SIRT3 regulates the metabolic transition of myofibroblasts in DN (Srivastava et al., 2021a). We also found a significant reduction in FGFR1 in the kidneys of DN rats, which is the same as that reported in the kidneys of diabetic mice; that is, endothelial-type FGFR1 deficiency contributes to fibrogenesis in DN (Li et al., 2020a). The effect was significantly improved after combined treatment with ursolic acid and insulin. In addition, regulation of NCAM/FGFR1 signaling inhibits the EMT program in human proximal tubular epithelial cells (Životić et al., 2018).

Currently, several miRNAs (e.g., miR-29, Let-7b, miR-21, miR-30b) have been found to be involved in the regulation of EMT and EndMT to affect disease processes such as renal fibrosis (Srivastava et al., 2019). GHOSH et al. (Ghosh et al., 2012) also confirmed that the TGF-β signaling pathway induced EndMT with altered expression levels of multiple miRNAs using miRNA array analysis. Subsequent studies have confirmed that miRNAs can affect renal fibrosis. For example, the miR-29 family directly targets and inhibits the expression level of Smad3, a molecule downstream of the TGF-β signaling pathway, thereby blocking the activation of the TGF-β pathway and inhibiting the EndMT and EMT processes (Chen et al., 2014).

The formation process of renal fibrosis is complex, and there are certain interactions between signaling pathways; therefore, multiple signaling pathways and their cross-relationships can be explored in future studies to gain a comprehensive understanding of the renal fibrosis process and to find the key intersections between signaling pathways, with the aim of finding the exact pathogenesis and the best treatment path for renal fibrosis.

## Conclusion

In conclusion, strict glycemic control plus ursolic acid could better reduce the degree of renal histopathological damage, apoptosis and fibrosis in rats. The underlying mechanism may be related to improving apoptosis and oxidative stress by regulating p38 MAPK, SIRT3, DPP-4 and FGFR1 levels, thereby blocking TGF-β signaling pathway activation and inhibiting EMT and EndMT processes.

## Data Availability

The original contributions presented in the study are included in the article/supplementary material, further inquiries can be directed to the corresponding authors.
